# Robust Adaptive Beamforming Algorithm for Sparse Subarray Antenna Array Based on Hierarchical Weighting

**DOI:** 10.3390/mi13060859

**Published:** 2022-05-30

**Authors:** Jian Yang, Xinxin Liu, Yuwei Tu, Weixing Li

**Affiliations:** 1 School of Engineering, Rocket Force University of Engineering, Xi’an 710025, China; neuqliuxinxin@163.com (X.L.); atywkk@163.com (Y.T.); 2 Science and Technology on Automatic Target Recognition Laboratory, National University of Defense Technology, Changsha 410073, China; lee_weixing@163.com

**Keywords:** array antenna, sparse subarray, digital beamforming, Covariance Matrix reconstruction

## Abstract

Sparse antenna arrays based on subarrays have more and more broad application prospects for the limitation of array space, real-time algorithm and hardware costs. Aiming at the beamforming technology of sparse antenna arrays based on subarrays, this paper proposes a robust adaptive beamforming algorithm based on hierarchical weighting. The algorithm performs conventional beamforming to calculate the weights of each element in the subarray, then the synthetic signals output by each subarray are used as sparse array metadata. The Interference-plus-Noise Covariance Matrix (INCM) is reconstructed by integration in two-dimensional space, and a convex optimization model of a multi-constraint array containing the signal pointing error was established to estimate the real guide vector. Finally, using the reconstructed INCM and the estimation of the guide vector, we obtain a weighted vector between the subarrays and output signal for the whole array. The simulation results show that the proposed algorithm has better Signal-to-Interference-and-Noise Ratio (SINR) and robustness compared with other algorithms for sparse subarray antenna array beamforming.

## 1. Introduction

Compared with the traditional single antenna, the array antenna can effectively realize the characteristics of narrow beam, low sidelobe, electronically controlled beam scanning, etc., and has been widely researched and applied in scientific fields, such as radar, speech processing, and wireless communication [1,2]. With the higher and higher requirements for the performance of the array antenna, the scale of the antenna array has continued growing, and the number of antenna elements even reaches tens of thousands or hundreds of thousands. One of the main factors to consider is how to reduce the hardware complexity and cost by reducing the number of antenna elements while ensuring the array antenna aperture becomes a hot issue in the research of array antenna technology. Especially in highly dynamic aircraft using phased array radar guidance, due to the limitation of carrier space and real-time algorithm, the antenna array is required not only to have larger array aperture, spatial resolution and anti-interference performance, but also to reduce the size of the array, the hardware complexity, the amount of data, the cost, and improve the real-time performance of the algorithm. For this reason, carriers, such as highly dynamic aircraft, often use a sparse array antenna design based on subarray structure, and its Digital Beamforming (DBF) technology becomes an important factor affecting the performance of the array antenna.

At present, robust adaptive beamforming algorithms are mainly divided into the following categories: Diagonal Loading (DL) [3,4], Eigenspace Beamforming (ESB) [5,6,7], the uncertainty set method [8,9,10], and the Interference-plus-Noise Covariance Matrix (INCM) algorithm, etc. The basic idea of the DL algorithm is to add a unit matrix of a certain magnitude to the covariance matrix to effectively suppress the steering error, but the magnitude of the added unit matrix is uncertain. ESB algorithm projects the assumed steering vector into the signal and interference subspace, estimates the real steering vector, and then calculates the weight vector of the adaptive beamformer by using the inverse of the sampling matrix. However, its performance deteriorates easily in the environment of low signal-to-noise ratio (SNR). The uncertain set method is to construct the constraint space according to the assumed interval of the desired direction and solve the real direction vector by using the convex optimization algorithm to effectively suppress the direction deviation. If the uncertainty interval is set reasonably, the algorithm has good performance, but the difficulty lies in obtaining the accurate range of the uncertainty interval, and when there is a large deviation in the direction of the expected signal, the performance will decrease significantly. Robust beamforming algorithm based on INCM reconstruction is a beamforming algorithm proposed by Gu et al., 2012. It uses Capon spatial spectrum estimation to reconstruct INCM and establishes a convex optimization model with quadratic constraints and quadratic rules to solve the robust adaptive beamforming algorithm of the desired signal steering vector [11]. In [12], a method is proposed to use the principle of maximum entropy power spectrum to estimate the power spectrum of the interfering signal and reconstruct the INCM by integrating. However, the algorithm cannot overcome the influence of array errors, such as array element position error, channel amplitude, and phase error. In order to improve the robustness of the integral reconstruction method under the condition of array error, a method of reconstructing the INCM by using Capon spatial spectrum estimation in the annular uncertainty set is proposed in [13]. Although the robustness of the algorithm under the condition of array error is improved, the computational complexity of the algorithm is greatly increased. Yang et al. used annular uncertainty sets to reconstruct INCM, and estimated array steering vectors through vector space projection, which further enhanced the robustness of the algorithm against array errors [14]. In [15], the steering vector and power estimation of interference signal are obtained by Capon spatial spectrum estimation in each angle sector of interference signal, and then the INCM is obtained by sparse reconstruction. The essence of this algorithm is the discrete representation of the method in [11], and its computational complexity increases with the increase in the number of interference signals. The differential reconstruction method mainly realizes the INCM reconstruction in a differential way by estimating the steering vector and power of the desired signal. In [16], a method is proposed to cancel the expected signal covariance matrix in a differential way from the Sample Covariance Matrix (SCM). Its essence is to replace the expected signal eigenvalue with the mean of the noise eigenvalue to reconstruct the INCM. The algorithm requires that the input SNR cannot be close to the Interference-to-Noise Ratio (INR), and the expected signal and interference can be effectively separated. The Oracle approximate shrinkage method is first used in [17] to estimate the covariance matrix of the array-received signal in a cyclic iterative manner on the basis of SCM, and then suppresses the desired signal component through differential cancellation to achieve INCM reconstruction. In [18], in order to improve the performance of adaptive beamforming in high SNR, the signal-free interference-plus-noise covariance matrix is reconstructed using MUSIC spatial spectrum as the power density distribution. However, how to improve the array performance under the error of the expected signal is not considered. In [19], for non-circular signals, three robust widely linear beamformers based on INCM reconstruction are proposed to cope with this problem of performance degradation at high SNR and high computational complexities. In [20], a robust adaptive beamforming algorithm is proposed based on a method for estimating the steering vectors and INCM reconstruction. In order to obtain the actual steering vectors, a subspace for each nominal steering vector in a small neighborhood is constructed firstly. Then, the adjusted steering vector can be found by searching along the gradient vector, which is orthogonal to the corresponding nominal steering vector neighborhood, to generate the highest Capon power amplitude. However, the method to get an adjusted steering vector is too simple and the estimation accuracy is limited. In [21], the proposed robust adaptive beamform based on INCM reconstruction uses a spare Bayesian learning algorithm to get high DOA accuracy and power estimation. Because of the superior DOA estimation accuracy of the spare Bayesian learning algorithm, the true steering vector estimation procedure is no longer necessary. In fact, this algorithm cannot completely overcome the direction error of the desired signal, which is an inevitable practical problem.

Aiming at the problem of estimating the direction of the desired signal under underdetermined conditions, array element sparseness is adopted. The sparse array refers to the array with the array element spacing greater than half a wavelength. The classical sparse arrays are coprime arrays and nested arrays [22]. Compared with conventional array antennae, sparse arrays have more spatial diversity. Under the same array aperture, sparse arrays require fewer array elements and have larger array apertures than uniform arrays [23]. Gu et al. used a one-dimensional linear coprime array to generate a virtual uniform array, and then used compressed sensing to compress the sampling and calculate the sample-compressed virtual covariance matrix to achieve adaptive beamforming [24]. In [25], Liu et al. expanded the ESB algorithm in the coprime array, used virtual array element expansion to construct the interference and noise subspace of the signal, and then estimated the steering vector of the signal and interference according to the projection method to realize the INCM reconstruction. In [26], a differential synthesis array was established, which used the sparse form to estimate the direction of arrival, and then reconstructed the INCM of the received signal to obtain the weighting vector. In [27], the proposed robust adaptive beamforming algorithm for the coprime array used an interpolated virtual ULA to obtain a high-precision DOA estimation, and further estimate the steering vectors and powers more accurately. Not just for the sparse arrays with specific formations, such as coprime arrays, nested arrays, etc., two robust adaptive beamforming algorithms based on INCM are proposed for a variety of sparse arrays in [28]. However, the error of the steering vector is not considered in detail.

At present, the INCM reconstruction algorithm is basically applied to one-dimensional uniform linear arrays, and the beamforming algorithm of two-dimensional planar array antennae using spatial spectrum to reconstruct INCM is rarely covered in the literature. In this paper, a robust adaptive beamforming algorithm based on hierarchical weighting is proposed for the robust adaptive beamforming technology of two-dimensional planar sparse subarray antenna array. Firstly, the weights of each element are calculated by fixed beamforming for each element in subarray. Then, the synthetic signals output by each subarray are used as sparse array metadata, and the INCM is reconstructed by integration in two-dimensional space. Meanwhile, the convex optimization model of the multi-constraint array, including signal pointing error, is established to estimate the real steering vector. Finally, the reconstructed INCM and the estimated guide vector are used to calculate the weighted vector between the subarrays, and the synthesized output signal of the entire array is obtained. The proposed algorithm conforms to the characteristics of the subarray-level sparse antenna array structure and improves the performance of the adaptive beamforming algorithm under this antenna array configuration.

## 2. Received Signal Model of Two-Dimensional Planar Sparse Subarray

### 2.1. Two-Dimensional Planar Uniform Sparse Array Signal Model

**Figure 1:**
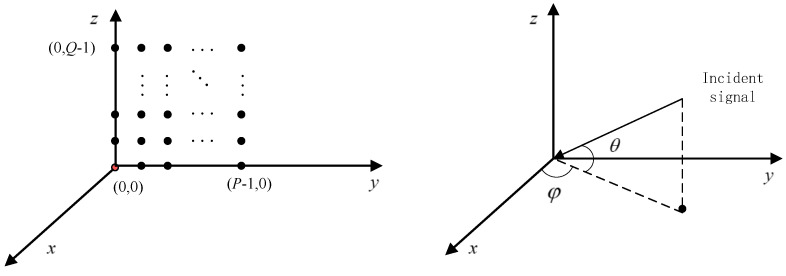
Structure of two-dimensional planar rectangular array antenna.

With the increasing requirements for radar systems, the two-dimensional planar array antenna is widely used. The array configuration of two-dimensional planar array antennae mainly include circular, L-shaped and rectangular arrays, etc. The general antenna structure of a two-dimensional rectangular planar rectangular array is shown in Figure 1. Assuming that the rectangular array antenna consists of P×Q array elements, the first array element in the lower-left corner is used as the reference array element (marked in red), and the position of the reference array element is (0,0). The position of each array element can be expressed as $\left(y_{p},z_{q}\right)$, that is

(1) $$\left\{\begin{array}{l} y_{p}=p\times d\\ z_{q}=q\times d \end{array}\right.$$

where, p=0,1,⋯, P−1, q=0,1,⋯,Q−1, and d are the array element spacing of a conventional array, meeting the requirement of d≤λ/2.

Assuming that the two-dimensional planar rectangular array antenna receives *D* signals, the incident direction of the signal is $\left(\varphi_{i},\theta_{i}),\;(i=0,1,\cdots ,D-1\right)$, $\varphi_{i}$ represents the azimuth angle, $\theta_{i}$ represents the elevation angle, where $\left(\varphi_{0},\theta_{0}\right)$ is the desired signal direction, the power of each signal is $\sigma_{i}^{2}\;(i=0,1,\cdots ,D-1)$, and the signals are not correlated with each other.

When the incident signal direction is $\left(\varphi_{i},\theta_{i}\right)$, the spatial phase difference between the (p,q)th array element and the reference array element can be expressed as

(2) $$\Delta \phi_{i}(p,q)=\frac{2\pi y_{p}\cos\theta_{i}\sin\varphi_{i}+2\pi z_{q}\sin\theta_{i}}{\lambda }$$

Then, the steering vector of the first row of array antennae along the positive y-axis can be expressed as

(3) $$a_{y}(\varphi_{i},\theta_{i})={\left[1,e^{-j2\pi y_{1}\cos\theta_{i}\sin\varphi_{i}/\lambda },\cdots ,e^{-j2\pi y_{p}\cos\theta_{i}\sin\varphi_{i}/\lambda }\right]}^{T}$$

Correspondingly, the steering vector of the first column array along the positive z-axis can be expressed as

(4) $$a_{z}(\varphi_{i},\theta_{i})={\left[1,e^{-j2\pi z_{1}\sin\theta_{i}/\lambda },\cdots ,e^{-j2\pi z_{q}\sin\theta_{i}/\lambda }\right]}^{T}$$

Therefore, the steering vector of a two-dimensional planar rectangular array antenna can be expressed as

(5) $$A_{i}=vec{\left(a_{y}(\varphi_{i},\theta_{i})a_{z}{\left(\varphi_{i},\theta_{i}\right)}^{T}\right)}^{T}$$

where, ${\left(\cdot \right)}^{T}$ denotes the transpose of the matrix, vec(⋅) denotes the stacking of the columns of the matrix into a single column vector.

When the two-dimensional planar rectangular array antenna receives all signals, its direction matrix can be expressed as

(6) $$A=[A_{0},A_{1},\cdots ,A_{D-1}]$$

where, the dimension of A is (P×Q)×D, and $A_{0}$ represents the steering vector of the desired signal.

At time *t*, the received signal is x(t)=As(t)+n(t), where s(t) is the spatial source signal, n(t) is Gaussian white noise, and s(t) can be expressed as

(7) $$s(t)={\left[s_{0}(t)\;s_{1}(t)\cdots \;s_{D-1}(t)\right]}^{T}$$

where $s_{0}$ represents the desired signal, and other signals $s_{1},\cdots ,s_{D-1}$ represent the interference signal. Assuming that the weight vector of receive beamforming is w, the output signal is $y(t)=w^{H}x(t)$, and the beam pattern is expressed as

(8) $$F(\varphi ,\theta )=w^{H}a(\varphi ,\theta )$$

where, a(φ,θ) represents the signal steering vector with an incident angle of (φ,θ), i.e., $a(\varphi ,\theta )=vec{\left(a_{y}(\varphi ,\theta )a_{z}{\left(\varphi ,\theta \right)}^{T}\right)}^{T}$. Generally, the beam pattern gain is expressed by Equation (10)

(9) $$G(\varphi ,\theta )=\frac{{\left|F(\varphi ,\theta )\right|}^{2}}{\max{\left|F(\varphi ,\theta )\right|}^{2}}$$

(10) $$G^{\prime }(\varphi ,\theta )=20\log G(\varphi ,\theta )$$

### 2.2. Two-Dimensional Planar Sparse Subarray Signal Model

**Figure 2:**
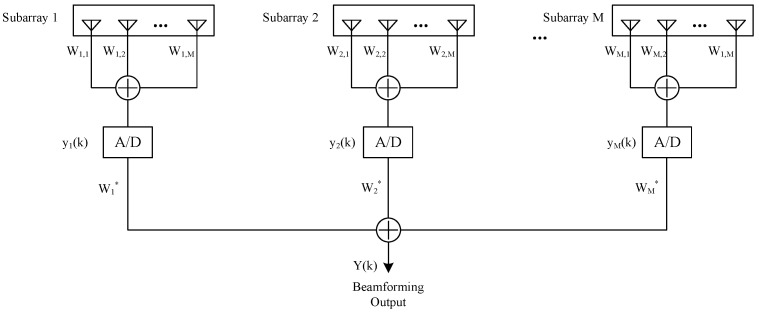
Schematic diagram of subarray beamforming.

Assuming that the two-dimensional sparse subarray consists of *M* subarrays, the fixed beamforming algorithm is used in the subarray to synthesize and output *M* channels of analog signals, and the signals are sent to *M* digital channels; then, the *M* channels of the signals are subjected to DBF to output 1 channel of digital signals. A schematic diagram of this is shown in Figure 2. Assuming that the two-dimensional plane sparse subarray receives *D* signals, the desired signal direction is $\left(\varphi_{0},\theta_{0}\right)$, the other *D*−1 signals are interference signals, and the angles are $(\varphi_{i},\theta_{i}),\;i=1,2,\cdots ,D-1$. Taking the m,m∈[1,M]th subarray as an example, the reference array element position is $\left(y_{m},z_{m}\right)$.

The steering vector of the first-row array along the positive y-axis in this subarray can be expressed as

(11) $$a_{y}(\varphi_{i},\theta_{i})={\left[e^{-j2\pi y_{m}\cos\theta_{i}\sin\varphi_{i}/\lambda },e^{-j2\pi (y_{m}+d)\cos\theta_{i}\sin\varphi_{i}/\lambda },\cdots ,e^{-j2\pi (y_{m}+(n_{y}-1)d)\cos\theta_{i}\sin\varphi_{i}/\lambda }\right]}^{T}$$

The steering vector of the first column array along the positive z-axis can be expressed as

(12) $$a_{z}(\varphi_{i},\theta_{i})={\left[e^{-j2\pi z_{m}\sin\theta_{i}/\lambda },e^{-j2\pi (z_{m}+d)\sin\theta_{i}/\lambda },\cdots ,e^{-j2\pi (z_{m}+(n_{z}-1)d)\sin\theta_{i}/\lambda }\right]}^{T}$$

Therefore, the steering vector of the mth subarray can be expressed as

(13) $$A_{i}=vec{\left(a_{y}(\varphi_{i},\theta_{i})a_{z}{\left(\varphi_{i},\theta_{i}\right)}^{T}\right)}^{T}$$

where, $A_{i}$ is a column vector with dimensions $n_{y}\times n_{z}$.

When the mth subarray receives all the signals, its direction matrix can be expressed as

(14) $$A(m)=[A_{0},A_{1},\cdots ,A_{D-1}]$$

where, the dimension of A(m) is $(n_{y}\times n_{z})\times D$.

At time *t*, the received signal of the *m* th subarray can be expressed as

(15) $$x_{m}(t)=A(m)s(t)+n_{m}(t)$$

where, s(t) is the spatial source signal, $s(t)={\left[s_{0}(t)\;s_{1}(t)\cdots \;s_{D-1}(t)\right]}^{T}$, where, $s_{0}$ represents the expected signal, and other signals $s_{1},\cdots ,s_{D-1}$ represents interference signal, $n_{m}(t)$ represents Gaussian white noise. Assuming that the weighted vector of the m-th subarray receiving beamforming is $w_{m}$, the received signal can be expressed as

(16) $$y_{m}(t)=w_{m}^{H}x_{m}(t)$$

Since the conventional beamforming algorithm is used in the subarray, according to Equation (13), the weight vector $w_{m}$ in the *m*th subarray can be obtained as

(17) $$w_{m}=A_{0}=vec{\left(a_{y}(\varphi_{0},\theta_{0})a_{z}{\left(\varphi_{0},\theta_{0}\right)}^{T}\right)}^{T}$$

After sampling, the output signal vectors of all subarrays can be expressed as

(18) $$y(k)={\left[y_{1}(k),y_{2}(k),\cdots ,y_{M}(k)\right]}^{T}$$

where, y(k) denotes the digital signal vector output by the subarray, k denotes sampled point, and the position of the reference array element of each subarray is $\left(y_{m},z_{m}\right)$; then, under the desired signal direction, the direction vector of the reference array element can be composed of subarray level guidance vector, and the subarray level steering vector can be expressed as

(19) $$a(\varphi_{0},\theta_{0})=\left[\begin{array}{c} e^{-j2\pi (y_{1}\cos\theta_{0}\sin\varphi_{0}+z_{1}\sin\theta_{0})/\lambda }\\ e^{-j2\pi (y_{2}\cos\theta_{0}\sin\varphi_{0}+z_{2}\sin\theta_{0})/\lambda }\\ \vdots \\ e^{-j2\pi (y_{M}\cos\theta_{0}\sin\varphi_{o}+z_{M}\sin\theta_{0})/\lambda } \end{array}\right]$$

Digital beamforming is then carried out between subarrays to output one signal. Assuming that the weighted vector among each subarray is w, the output signal of the two-dimensional plane sparse subarray can be expressed as

(20) $$Y(k)=w^{H}y(k)$$

## 3. Algorithm Introduction

The output Signal-to-Interference-and-Noise Ratio (SINR) of the beamformer can be expressed as

(21) $$\mathrm{SINR}=\frac{E\left\{w^{H}x_{0}(t)x_{0}^{H}(t)w\right\}}{E\left\{w^{H}x_{i+n}(t)x_{i+n}^{H}(t)w\right\}}=\frac{w^{H}R_{0}w}{w^{H}R_{i+n}w}=\frac{\sigma_{0}^{2}\left|w^{H}a(\varphi_{0},\theta_{0})\right|}{w^{H}R_{i+n}w}$$

where, $x_{0}(t)$ is the expected signal, $x_{i+n}(t)$ is the interference signal plus noise, $R_{i+n}$ represents the covariance matrix of interference plus noise, and $R_{i+n}$ can be expressed as

(22) $$R_{i+n}=\sum_{i=1}^{D-1}\sigma_{i}^{2}a_{i}a_{i}^{H}+\sigma_{n}^{2}I$$

where $\sigma_{i}^{2}$ and $\sigma_{n}^{2}$ are the energy of interference signal and noise, respectively. The design goal of the optimal beamformer is to maximize SINR. Therefore, the design method of the optimal beamformer is

(23) $$\underset{w}{\min}\;w^{H}R_{i+n}w\;s.t.\;w^{H}a(\varphi_{0},\theta_{0})=1$$

Accordingly, the weighting vector of the optimal beamformer can be expressed as

(24) $$w=\frac{R_{i+n}^{-1}a(\varphi_{0},\theta_{0})}{a{\left(\varphi_{0},\theta_{0}\right)}^{H}R_{i+n}^{-1}a(\varphi_{0},\theta_{0})}$$

Since $R_{i+n}$ cannot be obtained in practical applications, it is often replaced by the sample covariance matrix. The sample covariance matrix can be expressed as

(25) $$R_{0}=E[x(t)x(t)]=\delta_{0}^{2}a(\varphi ,\theta )a{\left(\varphi ,\theta \right)}^{H}+R_{i+n}$$

Therefore, Equation (23) can be further expressed as

(26) $$\underset{w}{\min}\;w^{H}R_{0}w\;s.t.\;w^{H}a(\varphi ,\theta )=1$$

The solution of the above equation is

(27) $$w=\frac{R_{0}^{-1}a(\varphi ,\theta )}{a{\left(\varphi ,\theta \right)}^{H}R_{0}^{-1}a(\varphi ,\theta )}$$

Under the condition of low SNR, $R_{0}$ is generally used to replace $R_{i+n}^{}$, and the beamforming weighting vector is obtained by Equation (27). However, when SNR increases, if $R_{0}$ continues to be used instead of $R_{i+n}^{}$ to calculate the weighting vector, there will be “self cancellation” of the desired signal, which will reduce the performance of the array antenna to receive the desired signal. Therefore, using the idea of spatial spectrum reconstruction $R_{i+n}^{}$ proposed in [11], this paper extends the INCM reconstruction method to the two-dimensional array model. Using Capon spatial spectrum in two-dimensional space, the INCM reconstruction is as follows

(28) $${\hat{R}}_{i+n}=\iint_{\theta ,\varphi \in \bar{\Theta }}\frac{a(\varphi ,\theta )a^{H}(\varphi ,\theta )}{a^{H}(\varphi ,\theta )R^{-1}a(\varphi ,\theta )}d\varphi d\theta$$

where $\bar{\Theta }$ is the complement of Θ, and Θ represents the angular region of the desired signal direction. If the INCM has been reconstructed and the desired signal direction is accurately known, the weighting vector can be expressed as

(29) $$w_{rec}=\frac{{\hat{R}}_{{}_{i+n}}^{-1}a(\varphi_{0},\theta_{0})}{a^{H}(\varphi_{0},\theta_{0}){\hat{R}}_{{}_{i+n}}^{-1}a(\varphi_{0},\theta_{0})}$$

where, $\left(\varphi_{0},\theta_{0}\right)$represents the expected signal direction.

In addition, in practical applications, the direction of the desired signal is often not known accurately, and there is an error in the estimation of the desired signal, so that the steering vector and the real steering vector are mismatched, resulting in the degradation of the array beamforming performance. Therefore, an optimization model is established to estimate the real steering vector.

First, the Signal-plus-Noise Covariance Matrix (SNCM) reconstruction can be expressed as

(30) $${\hat{R}}_{s+n}=\iint_{\theta ,\varphi \in \Theta }\frac{a(\varphi ,\theta )a^{H}(\varphi ,\theta )}{a^{H}(\varphi ,\theta )R^{-1}a(\varphi ,\theta )}d\varphi d\theta +\sigma_{n}^{2}I_{M}$$

where, $I_{M}$ represents the identity matrix of M×M.

In fact, according to the maximum output SINR criterion, assuming the possible target echo at the azimuth $\hat{\varphi },\hat{\theta }\in \Theta$, the Capon space spectrum estimation of the target echo signal in the angle sector can be expressed as

(31) $$P(\hat{\varphi },\hat{\theta })=\frac{1}{a{\left(\hat{\varphi },\hat{\theta }\right)}^{H}{\hat{R}}_{s+n}^{-1}a(\hat{\varphi },\hat{\theta })}$$

Then, relative to the real steering vector a(φ,θ), the spatial spectrum of the signal should meet

(32) $$\frac{1}{a{\left(\varphi ,\theta \right)}^{H}{\hat{R}}_{s+n}^{-1}a(\varphi ,\theta )}\geq P(\hat{\varphi },\hat{\theta })$$

Finally, the real steering vector can be decomposed into two components perpendicular to each other; one is a component parallel to a(φ,θ) and this component has no effect on the output interference plus noise energy relative to $a(\hat{\varphi },\hat{\theta })$, and the other component perpendicular to a(φ,θ) will affect Output interference plus noise energy. Therefore, according to Equations (31) and (32), the optimization model for solving a(φ,θ) can be expressed as

(33) $$\begin{array}{l} \underset{a}{\min}\;a^{H}{\hat{R}}_{i+n}^{-1}a\\ s.t.\;a^{H}{\hat{R}}_{s+n}^{-1}a\leq \;a{\left(\hat{\varphi },\hat{\theta }\right)}^{H}{\hat{R}}_{s+n}^{-1}a(\hat{\varphi },\hat{\theta })\\ \;{\left[a-a(\hat{\varphi },\hat{\theta })\right]}^{H}a(\hat{\varphi },\hat{\theta })=0 \end{array}$$

The optimization problem is a quadratic constrained quadratic programming problem, and both ${\hat{R}}_{i+n}^{-1}$ and ${\hat{R}}_{s+n}^{-1}$ are positive definite matrices. Therefore, the optimization model of Equation (33) is a convex optimization problem, which can be solved by the interior point method or optimization software, such as CVX [29].

After estimating the accurate steering vector, the weighted vector of the array can be expressed as

(34) $${\hat{w}}_{\mathrm{rec}}=\frac{{\hat{R}}_{{}_{i+n}}^{-1}a}{a^{H}{\hat{R}}_{{}_{i+n}}^{-1}a}$$

Therefore, the algorithm steps proposed in this paper are:

Step 1: According to the sparse subarray antenna array configuration, the array guidance vector A(m) is constructed according to Equations (11)–(14);
Step 2: According to the beam pointing requirements of the antenna array, calculate the weight vector $w_{m}$ of the antenna elements in the subarray according to Equation (17);
Step 3: Taking the beamforming output of each subarray as the sparse array element signal, ${\hat{R}}_{i+n}$ and ${\hat{R}}_{s+n}^{}$ are calculated according to Equations (28) and (30), respectively;
Step 4: According to the optimization model (33), estimate the true steering vector a(φ,θ);
Step 5: According to Equation (34), the weight vector ${\hat{w}}_{\mathrm{rec}}$ among the subarrays is obtained with ${\hat{R}}_{i+n}$ and the estimated true steering vector, and the final beamforming output result is obtained by using Equation (20).

## 4. Simulation Experiment Verification

**Figure 3:**
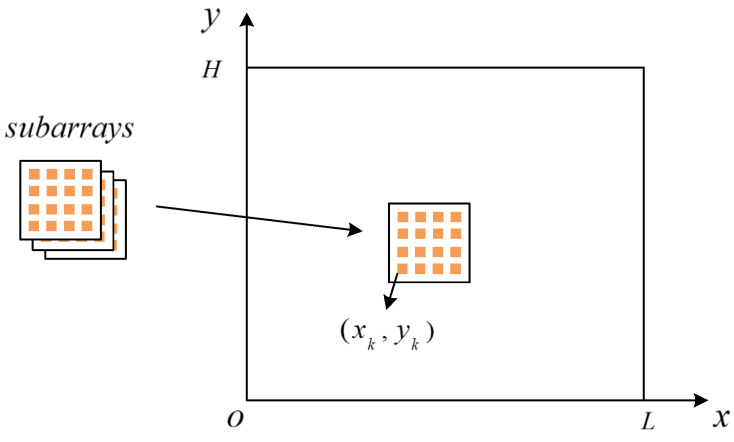
Sparse antenna array designed with eight regular subarrays.

It is assumed that the frequency of the desired signal is 15 GHz, the sparse antenna array consists of eight regular plane subarrays of 4 × 4 (four elements in each row and column), the array elements in the subarray are omnidirectional and equally spaced, and the spacing is d = 0.01 m. As shown in Figure 3, eight subarrays form a sparse antenna array on a plane, which can be designed by optimization methods, such as genetic algorithm and particle swarm algorithm [30]. For example, the positions of eight regular plane subarrays are shown in Table 1. X and Y are the abscissa and ordinate of the subarray positions, respectively, and the unit is meters.

Assuming that the azimuth and elevation angles of the desired signal direction are $\varphi_{0}=30^{\circ }$ and $\theta_{0}=10^{\circ }$, respectively, and the four interfering signal directions are ($30^{\circ }$,$40^{\circ }$), ($-20^{\circ }$,$0^{\circ }$), ($15^{\circ }$,$30^{\circ }$), and ($-10^{\circ }$,$10^{\circ }$), respectively, and INR = 20 dB, it is assumed that the azimuth angle sector in which the desired signal appears is $\left[\varphi_{0}-6^{\circ },\varphi_{0}+6^{\circ }\right]$ and the pitch angle sector is $\left[\theta_{0}-6^{\circ },\theta_{0}+6^{\circ }\right]$. In the simulation experiments, the comparison algorithms used include the LSMI algorithm in [31], the RAB-WCPO algorithm in [32], the REC-SPSS algorithm in [33], and the optimal beamformer (see Equation (24)). The loading value is 10-times the noise power in the LSMI algorithm, and the parameter ε=0.3 is used in the RAB-WCPO algorithm. For each simulation scenario, 200 Monte Carlo trails were performed.

### 4.1. Simulation for Array Beampattern

**Figure 4:**
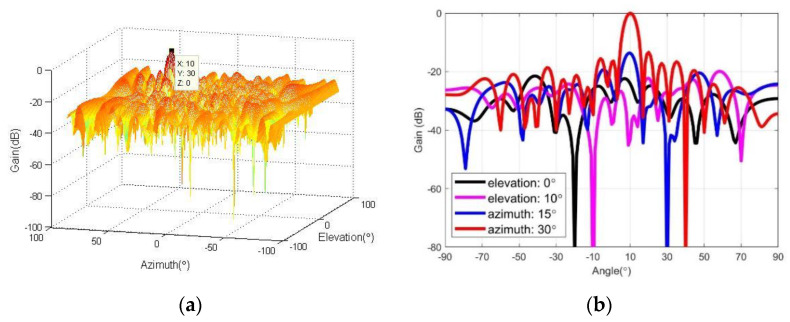
Array beampattern. (**a**) three dimensional beampattern; (**b**) section view of array beampattern.

Figure 4a shows the simulation results of the array beamforming pattern of the proposed algorithm. It can be seen that after the beamforming algorithm proposed in this paper, the direction of the main beam is consistent with that of the desired signal, the sidelobe is relatively low, there is no grating lobe, and the beam performance is good. Figure 4b is a section view in four angular directions, from which we can draw the following conclusions. (1) From the black line, when the elevation angular is $0^{\circ }$, there is a deep null at azimuth $-20^{\circ }$, which corresponds to the second interference signal of ($-20^{\circ }$,$0^{\circ }$). (2) From the magenta line, when the elevation angular is $10^{\circ }$, there is a deep null at azimuth $-10^{\circ }$, which corresponds to the fourth interference signal of ($-10^{\circ }$,$10^{\circ }$). (3) From the blue line, when the azimuth angular is $15^{\circ }$, there is a deep null at elevation $30^{\circ }$, which corresponds to the third interference signal of ($15^{\circ }$,$30^{\circ }$). (4) From the red line, when the azimuth angular is $30^{\circ }$, there are main beams at elevation $10^{\circ }$ and a deep null at elevation $40^{\circ }$, which correspond to the desired signal of ($30^{\circ }$,$10^{\circ }$) and the third interference signal of ($30^{\circ }$,$40^{\circ }$), respectively. The experimental results of beampattern simulation show that the proposed algorithm has correct main lobe beam direction, low side lobes and no grating lobes, and can adaptively form effective nulls in all interference signal directions.

### 4.2. Beamforming Performance without Direction Mismatch of the Desired Signal

In the second simulation, assuming that the desired signal direction is accurately known, the output SINR performance of the proposed algorithm and other beamforming algorithms is compared and analyzed under different input SNR and different snapshot numbers. In the performance comparison of output SINR versus input SNR, the input INRs and the number of snapshots were fixed as 40 dB and 1200. In the performance comparison of output SINR versus the number of snapshots, the input SNR and INRs were set as 10 dB and 40 dB, respectively.

**Figure 5:**
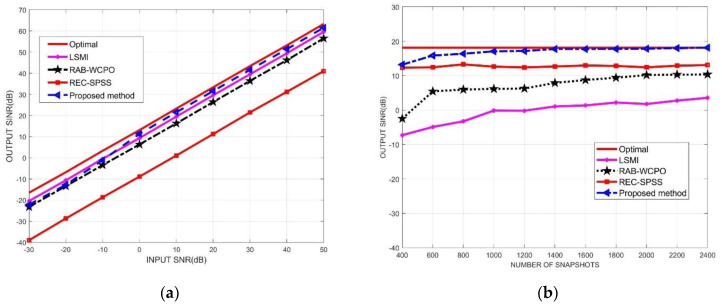
Output SINR of array antenna versus input SNR and number of snapshots, respectively. (**a**) output SINR versus input SNR; (**b**) output SINR versus number of snapshots.

As can be seen from Figure 5a, when the low SNR is less than −10 dB, the output SINR of the beamforming algorithm proposed in this paper is slightly worse than that of the LSMI algorithm, and better than the other two beamforming algorithms; when the SNR is greater than −10 dB, the output SINR of the beamforming algorithm proposed in this paper is better than other beamforming algorithms in comparison, and is closer to the ideal optimal beamformer. It is shown that the algorithm proposed in this paper can effectively solve the “self-cancellation” phenomenon of the desired signal when the signal-to-noise ratio increases, which verifies the effectiveness of the two-dimensional planar sparse subarray INCM reconstruction algorithm. As can be seen from Figure 5b, compared with other algorithms, the graph of the number of snapshots K and the SINR of the array output of the algorithm proposed in this paper has the fastest convergence speed, and is the closest to the optimal beamformer. The performance of the algorithm is better than other comparison algorithms.

### 4.3. Beamforming Performance with Direction Mismatch of the Desired Signal

In the third simulation, assuming that the desired signal direction contains errors, the output SINR performance of the proposed algorithm and other beamforming algorithms is compared and analyzed for different input SNR and different snapshot numbers. At this time, it is assumed that the errors of the azimuth and pitch angles of the desired signal and the actual values obey a uniform distribution in the range of $\left[-3^{\circ },6^{\circ }\right]$. In the performance comparison of output SINR versus input SNR, the input INRs and the number of snapshots were fixed as 40 dB and 1000. In the performance comparison of output SINR versus the number of snapshots, the input SNR and INRs were set as 10 dB and 40 dB, respectively.

**Figure 6:**
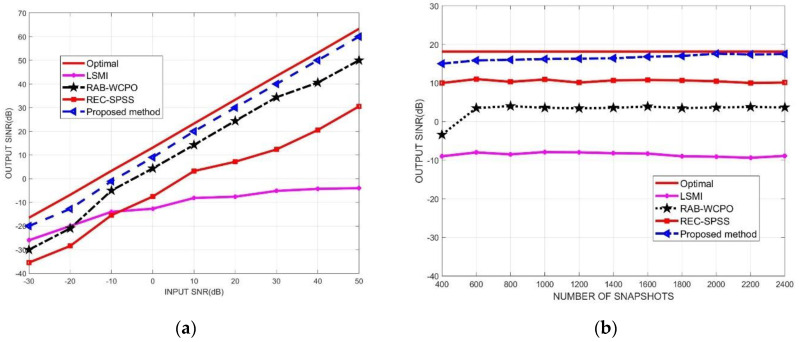
Output SINR of array antenna versus input SNR and number of snapshots, respectively. (**a**) output SINR versus input SNR; (**b**) output SINR versus number of snapshots.

From the simulation results in Figure 6a, it can be seen that under various input SNR conditions, the output SINR of the beamforming array proposed in this paper is better than other comparable algorithms, and is closer to the ideal optimal beamformer. From the simulation results in Figure 6b, it can be seen that under the conditions of different snapshot numbers, the output SINR of the beamforming array proposed in this paper is also better than other comparable algorithms, and is closer to the ideal optimal beamformer. The simulation results show that, compared with other algorithms, the algorithm proposed in this paper has better algorithm robustness when the desired signal direction contains errors, and the array beamforming performance is closer to the ideal optimal beamformer.

## 5. Conclusions

In this paper, a robust beamforming algorithm based on hierarchical weighting was proposed based on the structure characteristics of two-dimensional planar sparse subarray antenna array. Firstly, the weights of each element in the subarray were calculated by conventional beam weighting. Then, when calculating the adaptive weights between subarrays, the INCM reconstruction method was extended and applied to the two-dimensional array, and the INCM and SNCM were reconstructed by inter-partition double integration. At the same time, the Capon power spectrum and multi-constraint array convex optimization model, including expected pointing error, were used to estimate the real steering vector, and finally the inter-matrix weighted vector was obtained. The simulation results show that the beam direction of the proposed algorithm was consistent with the direction of the desired signal, and an effective null can be formed in the direction of the interference signal. Compared with other algorithms, the proposed algorithm has better beamforming performance, faster convergence speed, and better algorithm robustness for pointing errors.

## Figures and Tables

**Table 1:** The position of eight subarrays.

Position	Subarray 1	Subarray 2	Subarray 3	Subarray 4	Subarray 5	Subarray 6	Subarray 7	Subarray 8
X	0	0	0.0794	0.1325	0.1955	0.25	0.25	0.25
Y	0.0075	0.2349	0.1578	0.25	0.25	0.25	0.1339	0

## Data Availability

Not applicable.
